# A population-based survey of *FBN1* variants in Iceland reveals underdiagnosis of Marfan syndrome

**DOI:** 10.1038/s41431-023-01455-0

**Published:** 2023-09-08

**Authors:** Elin Ola Klemenzdottir, Gudny Anna Arnadottir, Brynjar Orn Jensson, Adalbjorg Jonasdottir, Hildigunnur Katrinardottir, Run Fridriksdottir, Aslaug Jonasdottir, Asgeir Sigurdsson, Sigurjon Axel Gudjonsson, Jon Johannes Jonsson, Vigdis Stefansdottir, Ragnar Danielsen, Astridur Palsdottir, Hakon Jonsson, Agnar Helgason, Olafur Thor Magnusson, Unnur Thorsteinsdottir, Hans Tomas Bjornsson, Kari Stefansson, Patrick Sulem

**Affiliations:** 1 Department of Pediatrics, Landspitali University Hospital, Reykjavik, Iceland; 2 deCODE Genetics/Amgen, Inc., Reykjavik, Iceland; 3 Faculty of Medicine, University of Iceland, Reykjavik, Iceland; 4 Department of Genetics, Landspitali Universtity Hospital, Reykjavik, Iceland; 5 Department of Cardiology, Landspitali University Hospital, Reykjavik, Iceland; 6 Institute for Experimental Pathology at Keldur, University of Iceland, Reykjavik, Iceland; 7 Department of Anthropology, University of Iceland, Reykjavik, Iceland; 8 McKusick-Nathans Institute of Genetic Medicine, The Johns Hopkins University School of Medicine, Baltimore, MD USA

**Keywords:** Disease genetics, Genetic testing

## Abstract

Marfan syndrome (MFS) is an autosomal dominant condition characterized by aortic aneurysm, skeletal abnormalities, and lens dislocation, and is caused by variants in the *FBN1* gene. To explore causes of MFS and the prevalence of the disease in Iceland we collected information from all living individuals with a clinical diagnosis of MFS in Iceland (*n* = 32) and performed whole-genome sequencing of those who did not have a confirmed genetic diagnosis (27/32). Moreover, to assess a potential underdiagnosis of MFS in Iceland we attempted a genotype-based approach to identify individuals with MFS. We interrogated deCODE genetics’ database of 35,712 whole-genome sequenced individuals to search for rare sequence variants in *FBN1*. Overall, we identified 15 pathogenic or likely pathogenic variants in *FBN1* in 44 individuals, only 22 of whom were previously diagnosed with MFS. The most common of these variants, NM_000138.4:c.8038 C > T p.(Arg2680Cys), is present in a multi-generational pedigree, and was found to stem from a single forefather born around 1840. The p.(Arg2680Cys) variant associates with a form of MFS that seems to have an enrichment of abdominal aortic aneurysm, suggesting that this may be a particularly common feature of p.(Arg2680Cys)-associated MFS. Based on these combined genetic and clinical data, we show that MFS prevalence in Iceland could be as high as 1/6,600 in Iceland, compared to 1/10,000 based on clinical diagnosis alone, which indicates underdiagnosis of this actionable genetic disorder.

## Introduction

Marfan syndrome (MFS) is an autosomal dominant disorder that typically affects the cardiovascular, ocular and skeletal systems. Other systems commonly involved are the skin, lungs and the dura mater [1]. Variation in expressivity is observed both between and within affected families [2]. The prevalence has been estimated at approximately 1/5,000–1/10,000, showing no gender, geographic or ethnic bias [3–5]. A prior (1996) Icelandic study using clinical criteria (albeit prior to Ghent nosology) estimated prevalence to be 1/15,000 [6]. Current standard of care for MFS patients involves treatment with beta-blockers (Propranolol, Atenolol, Metoprolol) and/or ARB inhibitors (Irbesartan, Losartan) as well as specific timing of surgery for aortic aneurysms, dependent on size of aneurysm [7]. Aorta dilatation, predisposition to aortic dissection, and valve deformities are the major cause of morbidity and early mortality in MFS. However, current treatment options have led to dramatically improved life expectancy for individuals with MFS [8, 9]. A clinical diagnosis is currently established according to the revised Ghent nosology [10], integrating clinical and genetic information.

MFS is caused by heterozygous pathogenic variants in the *FBN1* (*Fibrillin 1*) [11] gene on chromosome 15, which encodes a large protein of 2871 amino acids [12]. Identifying a pathogenic variant in *FBN1* in MFS patients allows for identification of at-risk family members and early diagnosis of individuals with limited symptoms during childhood and early adult years. Early diagnosis makes it possible to start preventive management and follow-up earlier, which may improve outcomes [13, 14]. *FBN1* is one of 78 genes that the American College of Medical Genetics (ACMG, version 3.1) recommends for reporting of incidental findings in clinical exome and genome sequencing, since MFS-causing variants in *FBN1* are considered to be an actionable genetic diagnosis [15]. Over 2000 variants in *FBN1* have been reported as pathogenic or likely pathogenic, of which 57% are predicted loss-of-function variants (i.e. frameshift, nonsense or at canonical splice acceptor and donor sites) and 43% are missense [16]. Whereas the vast majority of all predicted loss-of-function variants in *FBN1* (99%) classify as pathogenic or likely pathogenic according to ClinVar (April 2021), less than half (45%) of missense are classified as such, underscoring an interpretation challenge. In addition to MFS, certain variants in *FBN1* can cause other disorders. Some of these disorders have overlapping phenotypes with MFS, for example familial ectopia lentis and MASS syndrome. Other disorders, for example acromicric dysplasia, gelophysic dysplasia, stiff skin syndrome and Weill-Marchesani, have distinct phenotypes not typically observed in MFS, including short stature. For most of these syndromes, it seems to be the location of the pathogenic variant within *FBN1* that is critical for expressivity [17].

Variants in other genes have been known to cause Marfan-like phenotypes, such as variants in *TGFBR1*, *TGFBR2, TGFB2, TGFB3, SMAD2* and *SMAD3* causing Loeys-Dietz syndrome [18, 19], and variants in *FBN2* and *COL3A1* causing Congenital contractural arachnodactyly and Ehlers-Danlos syndrome type IV [20, 21]. Although variants in those genes cause phenotypes that overlap with phenotypes resulting from mutated *FBN1*, they are not currently thought to cause MFS and thus are not the focus of this work.

**Fig. 1:**
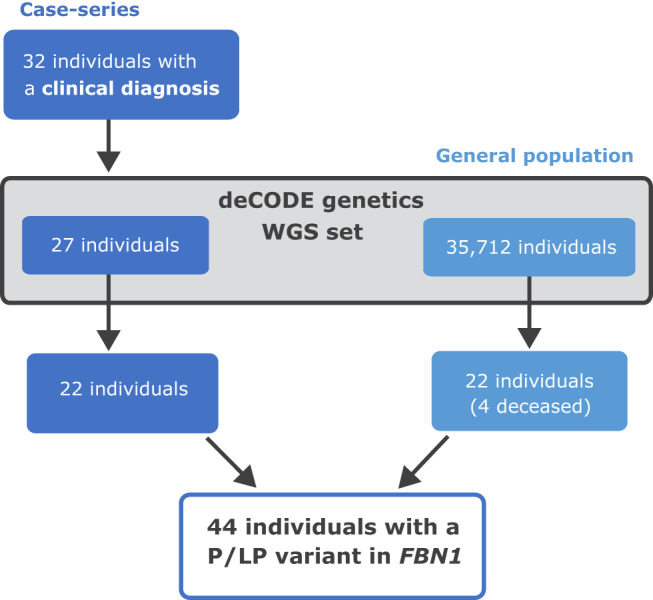
Study design to explore the prevalence of MFS in the Icelandic population. A diagram showing the study design. The study included individuals with a clinical diagnosis (case-series) and individuals found following a genotype-based approach using deCODE’s whole-genome sequencing (WGS) database representative of the general population.

We postulate that there may be a group of MFS cases who have eluded diagnosis in Iceland so we took a two-pronged approach (study design is highlighted in Fig. 1). First, we performed a case-series, based on discharge diagnoses of MFS (ICD10 Q87.4) and collected available clinical data in the Icelandic healthcare system. Secondly, we took a genotype-based approach, assessing all sequence variants in *FBN1* detected among 35,712 Icelanders whole-genome sequenced (WGS) at deCODE genetics (over 10% of the entire Icelandic population of 330 K) to search for potential MFS patients who have eluded diagnosis. Variants detected through WGS were also imputed into a total of 160,112 chip-genotyped Icelanders, increasing the power of detection of genotype-phenotype associations. This allowed us to identify potentially undiagnosed MFS individuals through genetic information. Here we present a nationwide study, reporting all *FBN1* variants detected in the Icelandic population, both in individuals with a clinical MFS diagnosis and those detected through a genotype-based approach. We provide a combined prevalence of clinically diagnosed and/or genotyped MFS in the Icelandic population, as well as describing a novel MFS-associated phenotype (abdominal aortic aneurysm (AAA)) based on cosegregation of a missense variant NM_000138.4:c.8038 C > T p.(Arg2680Cys) in a six-generation pedigree.

## Materials and methods

### Case series

Discharge diagnoses from Landspitali University Hospital and the only other major hospital system in Iceland (Akureyri hospital) were collected to search for all Icelandic patients with a clinical diagnosis of MFS (date of evaluation 04/07/2015). These diagnoses were achieved in many different ways and some predate the Ghent criteria. These two hospitals serve the vast majority of patients requiring complicated care. However, even though all these patients received a MFS discharge diagnosis from these hospitals, they may have received their initial diagnosis elsewhere and had follow-up care at other centers, e.g. in the general practice or at private clinics. Details of the signs and symptoms of some of these patients were therefore lacking, and additional phenotypic data were collected from family members or medical records when available. Data were analyzed for all of those with a diagnosis as well as individuals suspected to have the disorder. In addition, data from Landspitali University Hospital, death certificates and autopsy records were collected for ancestors and other family members to look for clues of the disorder. The oldest medical records viewed were from the late 19^th^ century. Patients were contacted, one from each family, and called in for an interview and a blood draw, although a detailed physical examination was not performed. Blood was also drawn from a few other undiagnosed family members.

### Deceased individuals with a MFS diagnosis

Data on deceased patients known to have had MFS were collected when available. Data from deceased individuals included in this study were collected from Landspitali University Hospital. These individuals were not included in final prevalence calculations.

### deCODE’s genealogical database

The book of Icelanders is a database that contains genealogical information for more than 900,000 Icelanders. It is estimated to contain data on the majority of the population since the settlement of the island, and 95% of Icelanders born after the year 1700. It has information on parents, dates of birth and death. Anonymous data from the database were used in this study, to establish pedigrees [22]. As this part of the study has no patient identifiers, clinical information is not available for those individuals apart from phenotype lists previously collected by deCODE.

### Identification of pathogenic and likely pathogenic *FBN1* variants

deCODE genetics has collected DNA samples for 160,112 Icelanders and 35,712 of those have undergone WGS (August 2017). Sequence variants in  exons and splice sites of the *FBN1* gene were assessed for all WGS individuals in the deCODE database. Variants with a minor allele frequency greater than 0.01% were excluded. On the individual basis, on WGS calls, limits were set on depth > 12 and allelic ratio > 0.25. Sanger sequencing was performed on DNA from close relatives within a single large family with a known pathogenic variant p.(Arg2680Cys).

Clinical significance of variants was obtained based on the ACMG’s guidelines for interpretation of sequence variants using the InterVar and VarSome websites [23, 24]. As previously described, variants in *FBN1* can cause other syndromes. If variants were reported to cause other syndromes in the ClinVar database they were excluded from the study. An association between the *FBN1* variants and common MFS clinical features was tested using logistic regression, treating clinical features as the response and allele counts for each variant as a covariate. The clinical features included height (increased height in carriers compared to non-carriers in the same family), severe thoracic aneurysm, mitral valve prolapse and congenital lens malformation. An association with these clinical features at a *p*-value < 0.05 was considered to be strong pathogenic evidence (ACMG criteria PS4) and was taken into account when following the ACMG guidelines for classification of sequence variants. Detailed ACMG classifications are listed in Supplementary Table 3. All variants reaching a “likely pathogenic” or “pathogenic” classification for MFS were included in the study. Association data were assessed in April 2021.

### Whole-genome sequencing and imputation

The methods used for WGS in deCODE have previously been described in detail [25]. Genotypes of close relatives of known MFS patients were predicted by imputation and then validated by targeted Sanger sequencing. Imputation is a method to infer genotypic status of variants that have not been directly tested via sequencing or chip-genotyping [26]. The method is based on the assumption that individuals sharing a haplotype over a certain region are more likely to carry the same variant in that region. deCODE genetics bases its imputation on long-range phasing of known haplotypes in the Icelandic population [27]. Variants identified through smaller sets of WGS individuals can be imputed into larger sets of chip-genotyped individuals, and increase power for phenotype-genotype interpretations.

### Calculating prevalence

We discuss all individuals carrying pathogenic or likely pathogenic variants, but when calculating prevalence, we limit to living individuals. The updated prevalence is calculated based on living carriers of *FBN1* variants predicted to be likely pathogenic or pathogenic for MFS. In the prevalence calculations we used the total population of Iceland on January 1st 2015 (329,100).

## Results

**Fig. 2:**
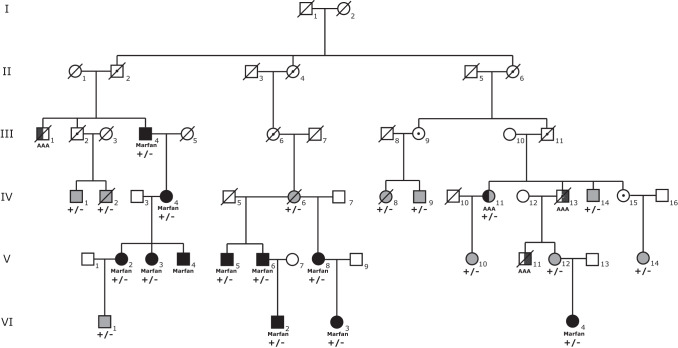
Pedigree presenting Family 1, with carriers of *FBN1* p.(Arg2680Cys). Full black circle/square (Marfan)=Clinically diagnosed MFS; Half circle/square=AAA; Circle/square with dot=obligate carrier; Full grey circle/square=Carrier without a known phenotype. +/−=Heterozygous carrier of NM_000138.4:c.8038 C > T p.(Arg2680Cys). AAA=Abdominal aortic aneurysm.

Our examination of medical records of individuals with a MFS discharge diagnosis (ICD-10 Q87.4) in the two major hospitals in Iceland yielded 32 living individuals with a clinical diagnosis of MFS, making the prevalence 1/10,000 in Iceland based on clinical diagnosis alone (Supplementary Table 1). We acquired biological samples from 27 of these 32 affected individuals and performed WGS. WGS yielded nine rare coding or splice site sequence variants in *FBN1* in 22/27 (81%) of these individuals (Table 1). In the remaining five individuals no pathogenic or likely pathogenic variant was identified, including in *FBN1, TGFBR1*, *TGFBR2, TGFB2, TGFB3, SMAD3, SMAD2, FBN2* and *COL3A1* (see WGS coverage over these genes in Supplementary Table 2). Out of the nine *FBN1* variants identified in our case-series, six have been previously reported as pathogenic or likely pathogenic (Table 1) whereas three are novel and classify as pathogenic or likely pathogenic based on the ACMG criteria (Methods). Four of the variants were private (i.e., carried by a single individual), four were carried by two closely related individuals, and one variant, p.(Arg2680Cys), was carried by 10 individuals, all of whom are part of the same extended family. There is one additional individual in the p.(Arg2680Cys) family with a MFS diagnosis for whom a DNA sample was not available to confirm the presence of p.(Arg2680Cys) (V-4 in Fig. 2). The p.(Arg2680Cys) variant is a known pathogenic variant, previously described as a recurrent de novo MFS variant [28, 29].

**Table 1:** All pathogenic and likely pathogenic *FBN1* variants detected in the study.

Nucleotide change (NM_000138.4)	Protein change (NP_000129.3)	*FBN1* exon	Carriers in case-series	^a^Family ID	Carriers in deCODE set	Inheritance	^b^Clinical significance	ClinVar ID	Previously published (ref)
***Variants detected in case-series***									
c.1464dupT	p.(Ile489TyrfsTer2)	12	1	Individ. 5	0	De novo	Pathogenic	-	No
c.1850G > A	p.(Cys617Tyr)	16	1	Family 4	0	De novo	Pathogenic	495563	Yes [43, 44]
c.2855-2 A > G	-	25	1	Individ. 8	0	Inherited	Pathogenic	1485695	^c^No
c.4211 A > G	p.(Asp1404Gly)	35	2	Family 6	0	Inherited	Likely pathogenic	-	Yes [45]
c.5788+5 G > A	-	47	2	Family 5	0	De novo	Pathogenic	42394	Yes [11, 34–36]
c.6446 A > G	p.(Tyr2149Cys)	53	1	Individ. 1	0	De novo	Pathogenic	42402	Yes [46, 47]
c.6744_6746delGGA	p.(Glu2248delGGA)	56	2	Family 3	0	Inherited	Likely pathogenic	-	No
c.7699+2 T > C	-	62	2	Family 2	0	Inherited	Pathogenic	-	No
c.8038 C > T	p.(Arg2680Cys)	64	10	Family 1	11	Inherited	Pathogenic	200127	Yes [27–29]
***Variants detected in a neonatal MFS patient, deceased***									
c.3290 G > A	p.(Cys1097Tyr)	27	1	Neonatal case	0	De novo	Pathogenic	549150	Yes [33]
***Variants detected in deCODE database***									
c.1616G > A	p.(Arg539Gln)	14	0	-	4	Inherited	Likely pathogenic	-	No
c.2860 C > T	p.(Arg954Cys)	25	0	-	4	Inherited	Pathogenic	495582	Yes [13, 48–50]
c.4085 C > G	p.(Thr1362Ser)	33	0	-	1	De novo	Likely pathogenic	-	No
c.7906 G > A	p.(Gly2636Ser)	64	0	-	1	NA	Likely pathogenic	393275	^c^No
c.8405 G > T	p.(Gly2802Val)	66	0	-	1	NA	Likely pathogenic	-	No

^a^A description of the families and individuals is provided in Supplementary Table 1.

^b^Clinical significance was obtained using the ACMG guidelines (see Supplementary Table 3 for ACMG criteria).

^c^Variant c.2855-2 A > G has been reported as Pathogenic on ClinVar, but not in the literature. Variant p.(Gly2636Ser) is reported as a variant of uncertain significance on ClinVar, is not reported in the literature.

**Fig. 3:**
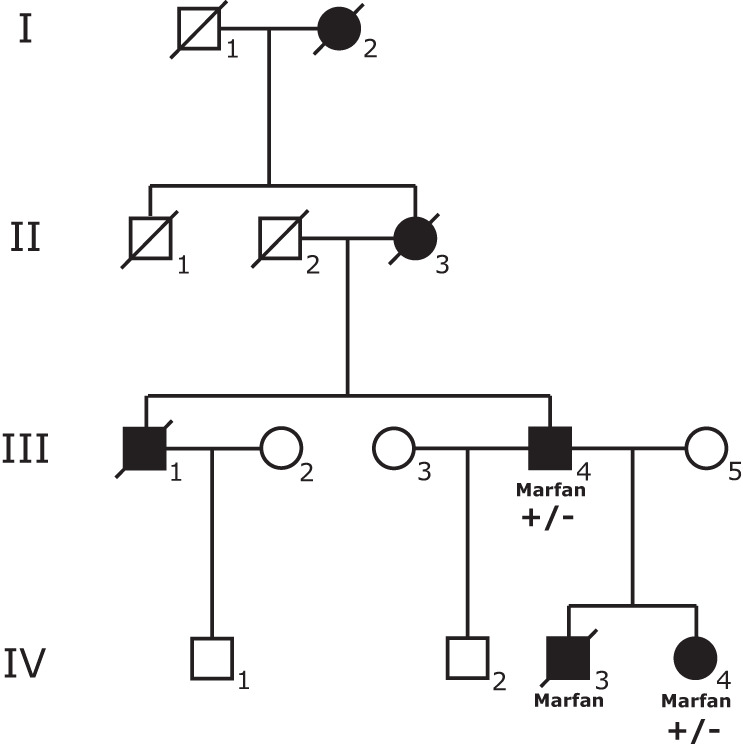
Pedigree presenting Family 2, with carriers of *FBN1* c.7699+2 T > C. Full black circle/square=Known thoracic aortic aneurym; Marfan=MFS clinical diagnosis; +/−=Heterozygous carrier of NM_000138.4:c.7699+2 T > C. Individual IV-3 died in a car accident at age 22. Individuals I-2, II-3, and III-1 all passed away because of a ruptured aortic aneurysm.

In addition to the 32 patients with a clinical diagnosis, there were two individuals with a clinical diagnosis who were deceased at the time of the study. One of them, a male infant, had a neonatal MFS presentation with severe symptoms, including a severe mitral valve defect which was detected prenatally and a MFS diagnosis that was made soon after birth. He was noted to have long extremities, thin stature, arachnodactyly, and other typical skeletal features. He had ectopia lentis and early myopia. He also had pulmonary bullae and spontaneous pneumothorax. His most severe signs included dilation of the aorta, enlarged heart and mitral valve prolapse. He passed away at 18 months of age, due to heart failure because of severe mitral valve regurgitation. A pathogenic variant was detected in this patient via genetic testing of *FBN1* prior to this study, NM_000138.4:c.3290 G > A p.(Cys1097Tyr) (Table 1). The second case was a 22-year-old male (individual IV-3 in Family 2, Fig. 3) who was diagnosed with MFS and had undergone surgery because of dilation of the ascending aorta in his teenage years, but passed away in an accident. He had two family members in the diagnosed case-series group, in whom we identified a splice donor variant in *FBN1* that classifies as pathogenic, NM_000138.4:c.7699+2 T > C (Table 1). Unfortunately no genetic material was available from individual IV-3 to assess the status of the c.7699+2 T > C variant in him. Interestingly this family has three other deceased family members who died at a young age (59, 22 and 27 years old) due to aortic aneurysm as stated in their death certificates. They did not have a diagnosis of MFS before their death, and additional clinical data were not available (Fig. 3).

We next expanded our study to the deCODE database of 35,712 WGS Icelanders and identified 61 very rare or novel, coding or splice sequence variants in *FBN1* (Supplementary Table 3). Six of the 61 variants classify as pathogenic or likely pathogenic based on ACMG criteria, one of which was previously detected in the case-series, p.(Arg2680Cys) (Table 1). In total, the six pathogenic or likely pathogenic variants are carried by 22 individuals. Two of these six variants have previously been reported as pathogenic by other groups, NM_000138.4:c.2860 C > T p.(Arg954Cys) and p.(Arg2680Cys), four are novel and classify as likely pathogenic based on ACMG criteria.

From the genotype-based approach we identified 11 additional carriers (not diagnosed with MFS) of p.(Arg2680Cys) in *FBN1*. These 11 carriers were all part of the same extended family as studied in the case-series. From the case-series we had identified 11 family members with diagnosed MFS, 10 of whom are confirmed carriers of p.(Arg2680Cys), amounting to 22 individuals within this six-generation family who are heterozygous carriers of p.(Arg2680Cys) and/or diagnosed with MFS. All 22 individuals are descended from a common ancestor couple, born in 1838 and 1841 (I-1 and I-2 in Fig. 2). The age of living family members who are carriers of p.(Arg2680Cys) ranges from 6 to 90 years. Affected family members have a wide range of clinical features, from isolated skeletal abnormalities to aortic dissection needing emergency surgery. As far as we know, no carrier has died due to MFS complications suggesting that heterozygosity for p.(Arg2680Cys) causes a relatively mild form of MFS. We have summarized available clinical features of individuals who are clinically diagnosed with MFS and/or are carriers of this variant (Table 2). This large kindred appears to have a notable predisposition to abdominal aneurysms. Four family members, including one carrier and one obligate carrier, had an abdominal aneurysm either diagnosed during life or based on post-mortem examination. On average the carriers’ height (*n* = 17) was 1.86 standard deviations over the population mean (around 12 cm taller), adding further support to the notion that this is a pathogenic sequence variant causing MFS [30].

**Table 2:** Clinical data of diagnosed MFS patients in the p.(Arg2680Cys) family and family members with an abdominal aortic aneurysm.

^a^Individual	Age	Sex	Ectopia lentis	Height SD	^b^Systemic score	Aortic thoracic involvement	Aortic abdominal involvement	Valve deformities
III-1	^†^78	M	NA	NA	NA	−	+	NA
III-4	90	M	+	2.58	NA	−	NA	−
IV-4	57	F	+	2.11	1	−	NA	AoR
IV-11	75	F	NA	1.50	NA	NA	+	NA
IV-13	^†^61	M	NA	NA	NA	NA	+	NA
V-2	35	F	−	NA	1	−	NA	ASA
V-3	33	F	+	2.22	2	+	NA	MVP
V-4	28	M	+	NA	2	+	NA	MVP
V-5	56	M	−	2.14	5	−	−	−
V-6	53	M	−	NA	1	+	+	NA
V-8	47	F	−	1.76	1	NA	NA	NA
V-11	^†^25	M	NA	NA	NA	−	+	NA
VI-2	7	M	NA	NA	NA	NA	NA	NA
VI-3	13	F	NA	NA	8	−	−	MVP
VI-4	20	F	+	1.31	10	−	NA	MVP

*M* Male, *F* Female, “+” indicates that clinical feature is present; “−” indicates that clinical feature hasn’t been observed, *NA* Data not available, *SD* Standard deviation, *AoR* Aortic valve regurgitation, *ASA* Atrial septal aneurysm, *MVP* Mitral valve prolapse.

^†^Individual is deceased.

^a^Number of the individual refers to his/her position in the pedigree in Fig. 2.

^b^For some individuals, clinical information was not available or limited, which can explain a low systemic score.

All in all, we have identified 15 pathogenic or likely pathogenic variants in *FBN1*, ten of which are missense variants, three are predicted to affect splicing, one is an in-frame deletion (3 bp), and one is a single base-pair duplication resulting in a premature termination codon. Three of variants showed an association with one or more clinical MFS features, providing further phenotypic support for the pathogenic nature of these variants (Supplementary Table 3). Seven variants were confirmed to be de novo variants, five of which were found in the case-series, NM_000138.4:c.1464dupT p.(Ile489TyrfsTer2), NM_000138.4:c.1850G>A p.(Cys617Tyr), NM_000138.4:c.2855-2 A > G, NM_000138.4:c.5788+5 G > A and NM_000138.4:c.6446 A > G p.(Tyr2149Cys). The splice region variant c.5788+5 G > A is de novo, shared by two siblings but absent from both of their parents based on WGS and Sanger sequencing of blood samples, indicating parental mosaicism. The sixth de novo variant is NM_000138.4:c.4085 C > G p.(Thr1362Ser), detected in a single individual through the genotype-based approach, and later determined to be de novo. The individual died at age 25, with no clinical data available, but the variant classifies as likely pathogenic based on ACMG criteria [31]. Lastly, the neonatal MFS case is a confirmed de novo case. Overall we identified 44 carriers of these 15 pathogenic/likely pathogenic variants in *FBN1*, 40 of whom were alive at the time of this study, and 22 of whom were previously diagnosed with MFS. In addition, we have knowledge of 10 individuals (32 with a clinical diagnosis minus the 22 with a known *FBN1* variant) with a clinical diagnosis of MFS from whom either no DNA was available, or no *FBN1* variant was detected. Based on these numbers, we estimate that the overall prevalence of MFS in Iceland could be as high as 1 in 6,600 (40 with a pathogenic/likely pathogenic variant in *FBN1* + 10 not genotyped MFS patients/without an identified *FBN1* variant, among 329,100 Icelanders).

## Discussion

We report a nationwide genetic study on MFS in Iceland, including all MFS patients with a clinical diagnosis as well as individuals identified through a genotype-based approach. Overall, we identified 15 pathogenic or likely pathogenic variants in *FBN1*. Seven are previously reported pathogenic or likely pathogenic variants and eight are new pathogenic/likely pathogenic variants. Our study thus expands the list of known MFS-causing variants and provides the healthcare system with a comprehensive list of variants in Iceland with emphasis on one highly represented variant p.(Arg2680Cys).

In this study we identified pathogenic *FBN1* variants in patients with both early and late presentations of MFS. We describe a single case of neonatal onset MFS in an infant with a de novo variant in *FBN1*; p.(Cys1097Tyr). The boy had a very serious clinical picture that led to his death at 18 months of age. The variant is located in exon 27, which belongs to a region with well-established genotype-phenotype correlation. Missense variants in exons 24-32 and variants causing skipping of exon 31 or 32 have been linked to severe phenotypes in all systems. Their location has been considered the best predictive factor in early onset dilation of the aorta, even when neonatal MFS is excluded [32]. The p.(Cys1097Tyr) variant has previously been reported to cause classical MFS in an adult [33].

Another variant we observed is a known recurrent de novo variant; c.5788+5 G > A, that is shared by two siblings in our sample set. This variant is one of the most frequent recurrent variants reported to cause classical MFS, with involvement of cardiovascular, ocular and skeletal systems [11, 34–36].

One of the variants we observed, NM_000138.4:c.6724 C > T p.(Arg2242Cys), is a previously reported pathogenic variant [37], but we observe no significant association with MFS phenotypes among 63 imputed heterozygous carriers in Iceland. Based on the evidence available to us, we classify p.(Arg2242Cys) as a likely benign variant and do not believe it has a role in MFS (Supplementary Table 3).

The most common pathogenic variant in our study, p.(Arg2680Cys), causes relatively mild symptoms among multiple carriers in a six-generation pedigree and does not appear to decrease fitness, unlike many of the sporadic variants we describe. p.(Arg2680Cys) is a known pathogenic variant reported multiple times as such [28, 29]. It has usually been described as mild, mainly affecting the skeletal and ocular systems, although one case presented with mitral valve prolapse without further involvement of the cardiovascular system [28]. In 2016, p.(Arg2680Cys) was found in compound heterozygous state in an individual with aorta dilation, together with NM_000138.4:c.4270 C > G p.(Pro1424Ala) in exon 35 of *FBN1*. Another family member who carried only p.(Arg2680Cys) presented with ectopia lentis and no other symptoms, at the age of two [29]. Here we report individuals, heterozygous for the p.(Arg2680Cys) variant, who have severe cardiovascular symptoms. In the Icelandic sample set we detected three patients carrying the variant who had dilation of the ascending aorta with one having had emergency surgery after aortic dissection. In addition, four carriers had mitral valve prolapse. Four individuals in this family have had AAAs, including one known carrier and one obligate carrier. The other two were close relatives where AAAs were observed post mortem, but no DNA was available to confirm a carrier status of p.(Arg2680Cys). AAAs in four individuals in this family is an apparent overrepresentation, since population prevalence of AAAs has been estimated to be around 1% [38]. We found no pathogenic or likely pathogenic variant in other genes previously linked to AAAs (HP:0005112) [39] in the one WGS family member with a confirmed AAA (individual IV-11 in Fig. 2). These data suggest that heterozygous carriers of p.(Arg2680Cys) should be screened for AAAs as part of their MFS workup. AAA is a known albeit rare clinical presentation in MFS, and AAAs associated with MFS are usually seen in younger individuals than AAAs in the general population (mostly in males after 65). The aneurysms also have different features, they are usually without mural thrombi, atherosclerotic changes are less frequent and abdominal aneurysms in MFS patients are more prone to rupture or dissection with a high mortality rate [40]. One of our p.(Arg2680Cys) carriers had an atrial septal aneurysm (ASA) without other cardiovascular signs, but ASAs have been associated with MFS before [41] so this may be another rare manifestation of p.(Arg2680Cys).

We applied a genotype-based method to search for MFS patients in Iceland, with the hope of gaining insight into possible underdiagnosis of the disease. A prior study on genotype-based identification of carriers of pathogenic variants using gnomAD data found that, in general, likely pathogenic variants in population datasets are more frequent than expected [42]. Moreover, they concluded that fibrillinopathies are likely underdiagnosed, and estimated that 1 in 5,000 individuals in the gnomAD dataset carry a pathogenic/likely pathogenic variant in *FBN1*, which is much higher than previous estimates of MFS prevalence [3–5, 42]. This is consistent with what we find in our genotype-based assessment of MFS in Iceland, i.e. that a substantial fraction of MFS patients are not identified as such, especially milder cases. Using a genotype-based approach does however have its limitations since there is a level of uncertainty on whether variants are truly pathogenic.

A limitation to our study is the small size of the Icelandic nation. With such a small population, slight changes in numbers of diagnosed MFS individuals will affect prevalence numbers greatly. Most noteworthy is the p.(Arg2680Cys) family that includes 22 family members who are either confirmed carriers of this pathogenic *FBN1* variant and/or diagnosed with MFS. Overall, this family accounts for around 50% of our cohort and has a great impact on the prevalence of MFS in Iceland. Furthermore, when calculating prevalence for the case-series, discharge diagnoses from the two largest hospitals in Iceland were used, and patients did not undergo clinical examination by the authors. Therefore, clinical information was limited for some of the individuals, as some patients were diagnosed outside of these two centers, and had their follow-up care elsewhere. Due to the lack of both MFS specialists and a specific MFS center in Iceland, it is unclear whether or not the Ghent criteria were applied for the diagnosis of the individuals in our cohort. We thus cannot exclude the possibility that some of these individuals were misdiagnosed, which could be skewing the prevalence based on clinical diagnosis. With this study, we wish to underscore the importance of obtaining a correct diagnosis of MFS patients, preferably with both a clinical diagnosis and identification of a pathogenic *FBN1* variant, to allow for accurate follow-up care of these individuals.

In summary, we describe a nationwide study on MFS in Iceland and provide an expansive list of disease-causing variants in *FBN1* in the Icelandic population. We describe a large kindred with mild MFS symptoms but a strong predisposition to the development of AAAs. We identified 32 individuals with a clinical diagnosis of MFS in Iceland, corresponding to a prevalence of approximately 1 in 10,000, which matches reported prevalence for MFS around the world [3–5]. By combining clinical data with genetic information, we show that the prevalence of MFS in Iceland could be higher than what is estimated from clinical diagnosis alone, or as high as 1 in 6600. Moreover, as we only have genetic information for less than half of the population, that number could still be an underestimate. Importantly, some of the individuals with a clinical diagnosis of MFS could be misdiagnosed, and it is possible that not all of the carriers of pathogenic/likely pathogenic *FBN1* variants identified through the genotype-based approach have MFS. However, MFS is a treatable genetic disorder that is life threatening if left untreated, and so finding these carriers is important for surveillance of ocular and vascular phenotypes based on current clinical guidelines.

### Supplementary information

Supplementary material

Supplementary table 1

Supplementary table 2

Supplementary table 3

## Data Availability

All data supporting the findings of this study are available within the main text of the article, and in the supplementary files. The pathogenic and likely pathogenic *FBN1* variants detected in our study have been submitted to ClinVar (https://www.ncbi.nlm.nih.gov/clinvar/, accession numbers: SCV003915948 - SCV003915961).
