# Supplementary material for: A population-based survey of FBN1 variants in Iceland reveals underdiagnosis of Marfan syndrome
Source: Eur J Hum Genet. 2023 Sep 8;32(1):44–51. doi: 10.1038/s41431-023-01455-0 (PMC10772070; doi:10.1038/s41431-023-01455-0)
Supplement: Supplementary file 1 — Supplementary material [file 41431_2023_1455_MOESM1_ESM.docx]

Supplementary to: **A population-based survey of *FBN1* variants in Iceland reveals underdiagnosis of Marfan syndrome**

Elin Ola Klemenzdottir^1,#^, Gudny Anna Arnadottir^2,3,#^, Brynjar Orn Jensson^2,#^, Adalbjorg Jonasdottir^2^, Hildigunnur Katrinardottir^2^, Run Fridriksdottir^2^, Aslaug Jonasdottir^2^, Asgeir Sigurdsson^2^, Sigurjon Axel Gudjonsson^2^, Jon Johannes Jonsson^3,4^, Vigdis Stefansdottir^4^, Ragnar Danielsen^5^, Astridur Palsdottir^6^, Hakon Jonsson^2^, Agnar Helgason^2,7^, Olafur Thor Magnusson^2^, Unnur Thorsteinsdottir^2,3^, Hans Tomas Bjornsson^1,3,4,8^, Kari Stefansson^2,3,*^, Patrick Sulem^2*^

^1^Department of Pediatrics, Landspitali University Hospital, Reykjavik, Iceland

^2^deCODE Genetics/Amgen, Inc., Reykjavik, Iceland

^3^Faculty of Medicine, University of Iceland, Reykjavik, Iceland

^4^Department of Genetics, Landspitali Universtity Hospital, Reykjavik, Iceland

^5^Department of Cardiology, Landspitali University Hospital, Reykjavik, Iceland

^6^Institute for Experimental Pathology at Keldur, University of Iceland, Reykjavik, Iceland

^7^Department of Anthropology, University of Iceland, Reykjavik, Iceland

^8^McKusick-Nathans Institute of Genetic Medicine, The Johns Hopkins University School of Medicine, Baltimore, MD USA

^#^Equal contribution

^*^Corresponding authors, equal contribution

Kari Stefansson, Email: kstefans@decode.is

Patrick Sulem, Email: patrick.sulem@decode.is

**Supplementary Tables:**

**Supplementary Table 1: Detailed clinical information for all diagnosed individuals with MFS.**

**External file: supplementary_table_1.xls**

**Supplementary Table 2: WGS coverage of *TGFBR1*, *TGFBR2*, *SMAD3*, *TGFB2*, *TGFB3*, *SMAD2*, *FBN2* and *COL3A1* in the six individuals without pathogenic/likely pathogenic variant in *FBN1*.**

**External file: supplementary_table_2.xls**

**Supplementary Table 3: All *FBN1* variants detected in the study, from case-series and deCODE database combined, in addition we present the nMFS case.**

Total of 70 variants in 164 individuals. 9 pathogenic/likely pathogenic variants were identified from the case-series. 61 variants were added by the genotype-based approach using deCODE genetics‘ database, of which 6 classify as pathogenic/likely pathogenic. Overall this table presents 15 pathogenic/likely pathogenic variants.

Clinical significance was obtained using the AMCG guidelines through the VarSome website. Variants in individuals with a clinical diagnosis are bolded.

**External file: supplementary_table_3.xls**

**Supplementary Figures:**

**
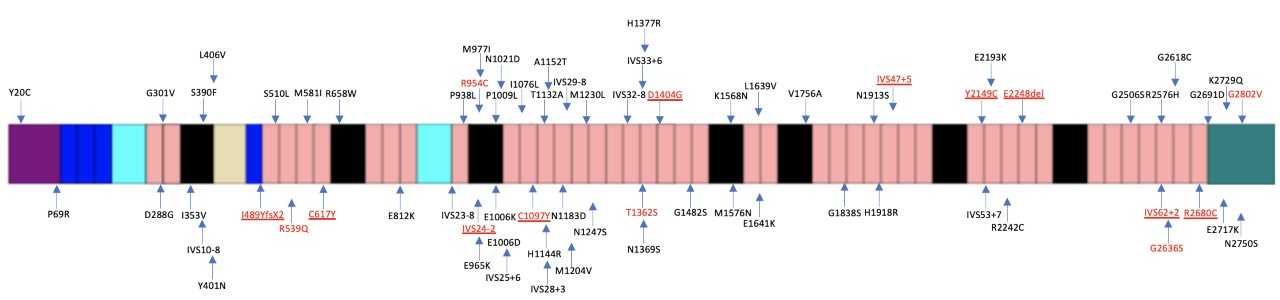
**

**Supplementary Figure 1:** **Domain organization of *FBN1* with variants detected in the Icelandic sample set.** Likely pathogenic and pathogenic variants are in red. Variants found in individuals with clinical diagnosis are underlined.
